# Health and well-being comparison between residents of serviced housing for older people and community-dwelling older adults in japan: a propensity score matching analysis

**DOI:** 10.1007/s00127-025-02947-8

**Published:** 2025-06-23

**Authors:** Hequn Wang, Kenjiro Kawaguchi, Ling Ling, Kazushige Ide, Atsushi Nakagomi, Katsunori Kondo

**Affiliations:** 1 Advanced Preventive Medical Sciences, Graduate School of Medical and Pharmaceutical Sciences, Chiba University, Chiba University, Inage-ku, Chiba, Chiba Japan; 2 Department of Social Preventive Medical Sciences, Center for Preventive Medical Sciences, Chiba University, Inage-ku, Chiba, Chiba Japan; 3 Department of Community Building for Well-being, Center for Preventive Medical Sciences, Chiba University, Inage-ku, Chiba, Chiba Japan; 4 Institute for Health Economics and Policy, Association for Health Economics Research and Social Insurance and Welfare, Minato-ku, Tokyo, Japan

**Keywords:** Physical health, Mental health, Social participation, Social interaction, Human flourishing

## Abstract

**Purpose:**

Serviced Housing for Older People (SHOP) in Japan offers barrier-free living environments and supportive services to enhance the health and well-being of older adults. This study aimed to compare the health and well-being of SHOP residents with community-dwelling older adults and to compare factors that may influence them.

**Methods:**

This cross-sectional study utilized propensity score matching to compare the health and well-being of 1,080 SHOP residents (69.4% female; mean age: 83.9 years) with 7,560 community-dwelling older adults (67.8% female; mean age: 84.1 years) from the Japan Gerontological Evaluation Study. Health and well-being were assessed using nine items. Additionally, social behaviours and social factors such as laughing frequency, regular outings, hobbies, depression, participation in preventive care activities, meeting friends frequently, emotional support, and eating with others, were compared.

**Results:**

SHOP residents exhibited significantly higher levels of happiness, life satisfaction, and physical health compared to community-dwelling older adults. They were more engaged in activities like regular outings, attending preventive care activities, meeting friends, and eating with others. They also reported higher frequencies of laughter and received emotional support.

**Conclusion:**

SHOP may improve the health and well-being of older adults. These findings can help in developing age-friendly housing initiatives to address the challenges of an aging society.

**Supplementary Information:**

The online version contains supplementary material available at 10.1007/s00127-025-02947-8.

## Introduction

The global population is aging rapidly, with projections suggesting that one in six individuals will be aged 65 or older by 2050 [1]. This demographic transition poses significant challenges for public health systems, particularly in promoting and maintaining the health and well-being of older adults. In response to this global aging trend, the World Health Organization introduced the Age-Friendly Cities and Communities initiative to create environments that promote the health and well-being of older adults [2]. Housing is a crucial element of this initiative and plays a crucial role in promoting the health and well-being of older adults. By providing safe, comfortable, and accessible environments, well-designed housing can enhance independence, facilitate social cohesion and engagement, and contribute to age-friendly development [3–5].

Globally, senior housing models in countries like the Netherlands and Finland have shown promise in promoting social connections among older adults through features like public spaces, communal dining, and group activities, thereby enhancing the health and well-being of older adults [6, 7]. For instance, in Central Finland, a midsized town hosts the senior housing complex with accessible, low-maintenance apartments, ample common areas for social interaction, and a convenient location near green spaces, essential services, and public transportation, fostering social engagement [6]. In the United States, older adults residing independently in continuing care retirement communities who actively engage in community-organized social activities have been found to experience a significantly slower decline in quality of life [8]. In Japan, where 29.1% of the population is aged 65 years or older [9], the demand for age-friendly housing is pressing. The Serviced Housing for Older People (SHOP) system was established under the “2011 Act on Securement of Stable Supply of Elderly Persons’ Housing” [10]. SHOP is a rental housing system with barrier-free environments and support services, including safety monitoring, daily consultations, and staff assistance with daily activities, enabling residents to maintain independence and enhance their health and well-being [11]. Most SHOP residents are older adults living alone or with a spouse.

Research on senior housing, including SHOP, suggests potential benefits for the health and well-being of older adults. Evidence suggests that senior housing residents often report better self-rated health [12] and higher levels of social participation [13] compared to community-dwelling older adults. Furthermore, participation in organized social activities in senior housing has been linked to a slower decline in quality of life over time [8]. However, research from Finland indicates that senior housing residents, particularly men, may experience lower physical function compared to community-dwelling older adults [14]. Additionally, relocation to senior housing can negatively impact the mental well-being and physical performance of older adults [15–17]. These conflicting findings highlight the need for further exploration, as the impact of senior housing on health and well-being remains controversial.

Although existing studies provide valuable insights, there is a lack of direct comparisons between the health and well-being of senior housing residents, including those in SHOP, and community-dwelling older adults [18]. Furthermore, the social and environmental factors within senior housing that may enhance health and well-being, such as social participation, physical activity, and emotional support, have not been fully explored. This study aimed to compare SHOP residents with community-dwelling older adults to better understand the potential benefits of senior housing for older adults’ health and well-being.

## Methods

### Study design

This cross-sectional study utilized propensity score matching (PSM) to compare the health, well-being, and factors associated with health and well-being between SHOP residents and community-dwelling older adults in Japan.

### SHOP group

Participants were recruited from 39 SHOP facilities named “Grandmast,” which cater primarily to older adults who can live independently. These facilities are operated by Sekisui House Real Estate Tokyo, Ltd., and are located in urban areas across nine prefectures in Japan, including Tokyo, Kanagawa, Saitama, Chiba, Kyoto, Aichi, Osaka, Hyogo, and Nara. These facilities are rental properties specifically designed for older adults who can live independently, without routine caregiving or medical services. Residents are generally in good health, financially stable, and capable of managing daily activities. In contrast to other types of SHOPs in Japan, which may cater to residents with varying care needs, Grandmast targets a more independent demographic. Each facility typically offers one- or two-bedroom units (approximately 45 and 60 m², respectively), accommodates approximately 60 residents on average, and has a mean building age of 8.7 years. Monthly housing costs usually consist of ¥180,000, ¥30,000, and ¥50,000–¥80,000 (excluding tax) for rent, maintenance, and lifestyle support services, respectively. To enhance residents’ health and well-being, Grandmast provides various services, including a communal dining area offering nutritionally balanced meals, weekly chair-based exercise classes, and concierge support for transportation, deliveries, daily living consultations, and referrals to external services (e.g., cleaning and caregiving). Between February and March 2023, a self-administered questionnaire was distributed to 1,700 residents. The survey collected data on demographics, health status, and well-being. Out of the 1,108 respondents (response rate: 65.2%), individuals under the age of 65 (*n* = 17) and those who did not provide consent (*n* = 6) were excluded from the analysis. The final sample (Supplementary Fig. 1) for the analysis included 1,085 participants, with a mean age of 83.9 ± 6.5 years. These participants were older adults living alone or with one other person, typically a spouse or child.

### Control group: community-dwelling older adults

Data for the control group were collected from the Japan Gerontological Evaluation Study 2022 wave, a nationwide longitudinal survey focusing on the social determinants of healthy aging among functionally independent individuals aged 65 years in Japan [19]. The survey was conducted through a self-administered mail questionnaire from November to December 2022, targeting 338,742 individuals aged 65 years in 75 municipalities. A total of 227,731 respondents participated in the survey, resulting in a response rate of 66.2%.

After excluding 24,530 participants who did not provide consent and 158,091 participants residing in non-designated cities, 45,110 participants from ordinance-designated cities, defined as cities with populations of 500,000 or more [20], were included in the analysis as SHOP is located in such cities. Subsequently, 372 individuals with missing gender information and 22,389 participants without health and well-being data (as they did not complete the relevant survey items) were excluded, leaving 22,349 eligible participants. To match the sample characteristics of SHOP residents, who are older adults living alone or with one other person, 6,700 participants from households with three or more members were excluded. The final analytical sample (Supplementary Fig. 1) comprised 15,649 participants, with a mean age of 76.3 ± 6.5 years.

### Outcome measure: health and well-being

Health and well-being were primarily assessed using the Human Flourishing Framework, a widely used multidimensional instrument with excellent internal consistency (Cronbach’s alpha = 0.89–0.93) [21]. We selected eight items across four domains relevant to aging populations: happiness and life satisfaction, mental and physical health, life meaning and purpose, and close social relationships, based on their relevance to aging populations and their significance in prior well-being studies [22]. An additional culturally relevant item on Ikigai—a concept reflecting life purpose and meaning in Japanese culture—was added to the life meaning and purpose domain, based on previous studies among older Japanese adults [23, 24]. Nine questions were utilized to assess these domains, with each question scored independently on a scale from 0 (worst state) to 10 (best state), where higher scores indicated better health and well-being. Happiness and life satisfaction were assessed with the following questions: “How happy are you at present?” and “Overall, are you satisfied with your current life?” Mental and physical health were assessed using the following questions: “How would you rate your physical health?” and “How would you rate your mental health?” Life meaning and purpose were evaluated using the following questions: “Overall, do you feel that what you do in your life is worthwhile?” “I have something to live for (Ikigai).” and “I understand my life purpose.”, Close social relationships were measured using the following questions: “I think I have satisfactory and desired relationships.” and “I am satisfied with my friendships and relationships.”

### Social behaviours and social factors associated with health and well-being

Based on previous research, various social behaviours and social factors were compared between the two groups: frequency of laughter (almost daily) [25], regular outings (at least five times a week) [26], engagement in hobbies [27], participation in preventive care activities (such as health exercise programs or group events) [28], meeting with friends frequently (at least four times a week) [29, 30], receiving emotional support (having someone to listen to their complaints and worries), and opportunities for eating with others (at least once a week) [31]. Given the negative impact of depression on well-being [31], we compared average depression scores between the two groups using the 15-item Geriatric Depression Scale (GDS-15). Scores range from 0 to 15, with higher scores indicating more severe depressive symptoms. The GDS-15 has demonstrated high internal consistency (Cronbach’s alpha > 0.80) and validity in older Japanese populations [32].

### Covariates

Based on a previous study on senior housing [14], 12 demographic and health-related variables were selected as covariates for the PSM model. The covariates included gender (male, female), age (continuous value), education (categorized as less than 10 years, 10–12 years, 13 years or more, and other), activities of daily living (respondents were asked “do you need someone to care for you in your daily life?” and classified as “not need care/assistance” if they answered “do not need care/assistance,” “need but not have care/assistance” if they answered “need some kind of care/assistance but do not currently receive it,” and “need and have care/assistance” if they answered “need of care/assistance and receiving care/assistance”), equivalent income (continuous value), living arrangement (live alone or living with others), assets (less than 10 million yen, 10–50 million yen, 50 million yen or more), employment status (employed or unemployed), body mass index (continuous value), diseases in treatment (have or not have), self-rated health (operationalized based on responses to the question “how is your current health condition?,” with responses of “excellent” or “good” categorized as “good” and responses of “fair” or “poor” categorized as “poor”), and degree of need for care (independence or need care).

### Statistical analysis

The PSM analysis was conducted to compare outcomes and associating factors between the SHOP and control groups. Prior to the analysis, missing data for all variables were imputed using the missRanger package in R [33], which applies random forest methods for robust imputation. Propensity scores for SHOP residency were computed through probit regression analysis, and matching was performed using nearest neighbor matching with replacement at a 1:7 ratio. The quality of the matching process was assessed by calculating the absolute standardized differences, with values below 0.10 indicating adequate balance [34]. Following matching, the Wilcoxon rank sum test was employed to compare health and well-being outcomes between the two groups, with statistical significance set at a *p*-value less than 0.05. Chi-square tests and Wilcoxon rank sum tests were applied to assess differences in supporting factors between the groups post-matching. To evaluate the robustness of between-group differences, we conducted sensitivity analyses for each of the nine health and well-being outcomes. In each model, we included the remaining eight outcomes as covariates, recognizing their conceptual and empirical interrelatedness. These analyses intended to assess whether the focal outcome remained considerably different between groups after controlling for correlated well-being indicators. A Bonferroni correction was applied to adjust for multiple testing, setting the statistical significance threshold at a *p*-value less than 0.0056 (0.05/9). All analyses, except for data imputation in R software, were conducted using Stata/SE version 18.0 (Stata Corp, College Station, TX, USA).

## Results

Table 1 summarizes the baseline characteristics of the SHOP and control groups, with higher percentages shown in bold. Compared to the control group, the SHOP group had a higher proportion of females (69.4% vs. 52.5%), older individuals (mean age: 83.9 years vs. 76.3 years), those with higher education levels (≥ 12 years: 49.4% vs. 41.7%), individuals living alone (87.8% vs. 26.8%), unemployed individuals (95.2% vs. 71.3%), and a higher prevalence of diseases under treatment (88.9% vs. 81.5%). The control group exhibited better health status (84.6% vs. 81.9%) and greater independence (95.1% vs. 62.2%) than the SHOP group.

**Table 1:** Characteristics of the control group and the SHOP^a^ group before propensity score matching

		Control group	SHOP group	Total
		*n* = 15,649 (93.5%)	*n* = 1,085 (6.5%)	*n* = 16,734 (100.0%)
Sex	Female	8,213 (52.5%)	**753 (69.4%)**	8,966 (53.6%)
Age		76.3 (6.5)	**83.9 (6.5)**	76.8 (6.7)
Education (years)	<10	2,584 (16.5%)	107 (9.9%)	2,691 (16.1%)
	10 ~ 12	6,367 (40.7%)	431 (39.7%)	6,798 (40.6%)
	> 12	6,522 (41.7%)	**536 (49.4%)**	7,058 (42.2%)
	Other	176 (1.1%)	11 (1.0%)	187 (1.1%)
Equivalent income (million yen)		2.52 (1.71)	2.48 (1.55)	2.51 (1.70)
Living arrangement	Living alone	4,188 (26.8%)	**953 (87.8%)**	5,141 (30.7%)
Employment status	Unemployed	11,159 (71.3%)	1,033 (95.2%)	12,192 (72.9%)
Assets (million yen)	<1	1,580 (10.1%)	220 (20.3%)	1,800 (10.8%)
	1 ~ 5	1,992 (12.7%)	62 (5.7%)	2,054 (12.3%)
	5 ~ 10	2,156 (13.8%)	72 (6.6%)	2,228 (13.3%)
	10 ~ 50	6,765 (43.2%)	545 (50.2%)	7,310 (43.7%)
	>50	3,156 (20.2%)	186 (17.1%)	3,342 (20.0%)
Activities of Daily Living (ADL)	Not need care/assistance	14,378 (91.9%)	805 (74.2%)	15,183 (90.7%)
	Need but not have care/assistance	921 (5.9%)	127 (11.7%)	1,048 (6.3%)
	Need and have care/assistance	350 (2.2%)	153 (14.1%)	503 (3.0%)
Body Mass Index (BMI)		22.5 (3.1)	21.5 (3.0)	22.5 (3.1)
Self-rated health	Good	**13**,**240 (84.6%)**	889 (81.9%)	14,129 (84.4%)
Diseases in treatment	Have	12,760 (81.5%)	**965 (88.9%)**	13,725 (82.0%)
Degree of need for care	Independent	**14**,**878 (95.1%)**	675 (62.2%)	15,553 (92.9%)

^a^ SHOP: Serviced Housing for Older People

**Fig. 1:**
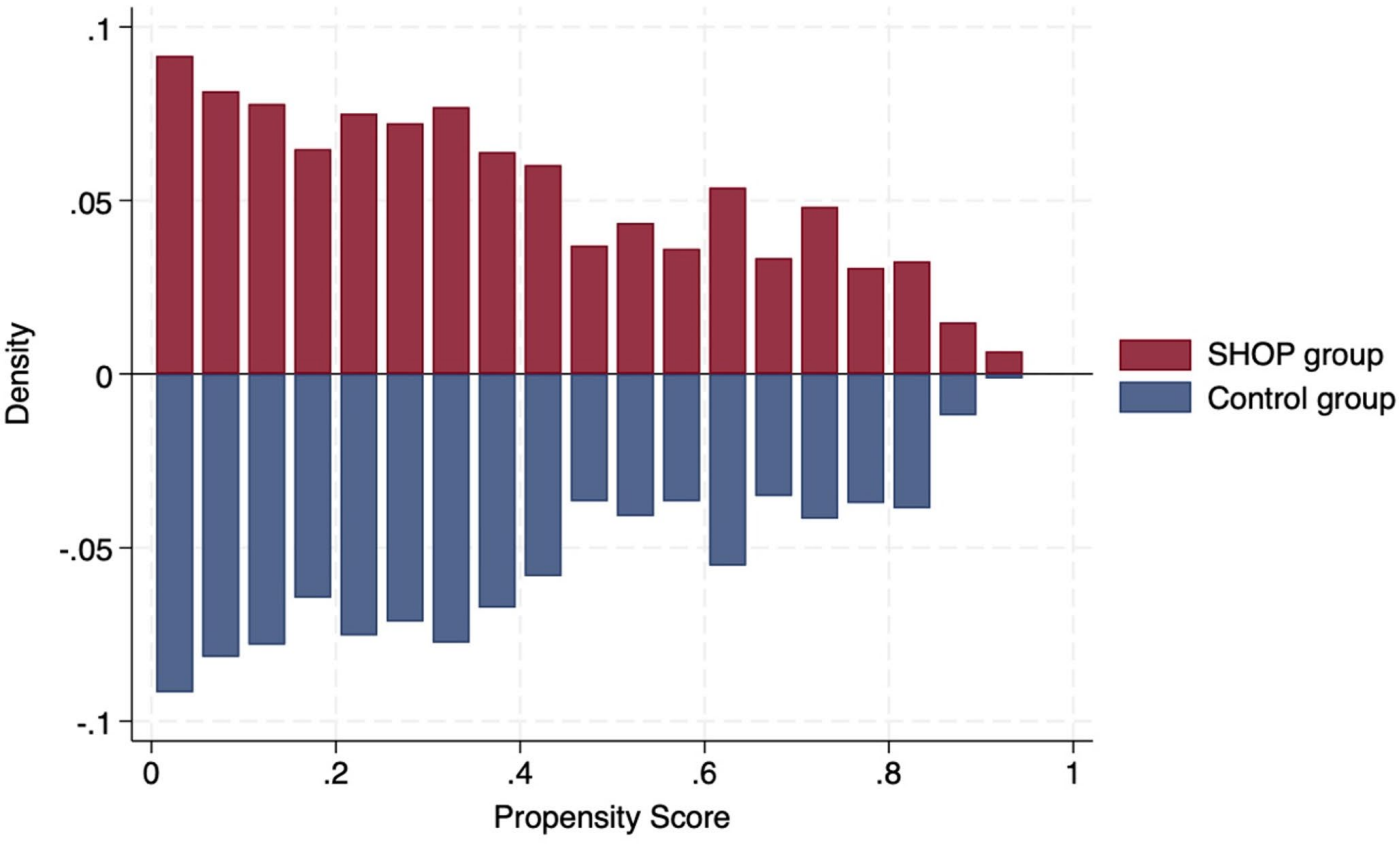
Distribution of propensity scores between the SHOP group and the control group after propensity score matching. SHOP: Serviced Housing for Older People

In the PSM analysis, a 1:7 matching ratio was applied, resulting in 1,080 individuals in the SHOP group and 7,560 in the control group. Among matched control group participants, 74% individuals reported owning their homes, and 19% lived in rental housing (based on available cases). As shown in Fig. 1, the distribution of propensity scores between the two groups became more comparable after PSM compared to before matching (Supplementary Fig. 1).

Table 2 presents the characteristics of the SHOP and control groups after PSM. All covariates had absolute standardized difference values below 0.10, indicating similar covariate distributions between the two groups.

Table 3 summarizes the mean scores of health and well-being for participants in the SHOP and control groups, with higher mean scores and *p* < 0.05 shown in bold. Participants in the SHOP group exhibited significantly higher mean scores in happiness (*p* < 0.001), life satisfaction (*p* < 0.001), and physical health (*p* < 0.001) compared to the control group. No significant differences were observed for the remaining items.

**Table 2:** Characteristics of the control group and the SHOPa group after propensity score matching

		Control group	SHOP group	Total	SD
		*n* = 7,560 (87.5%)	*n* = 1,080 (12.5%)	*n* = 8,640 (100.0%)	
Sex	Female	5,036 (66.6%)	750 (69.4%)	5,786 (67.0%)	0.061
Age		84.0 (6.6)	83.9 (6.5)	84.0 (6.6)	−0.02
Education (years)	< 10	771 (10.2%)	104 (9.6%)	875 (10.1%)	−0.019
	10 ~ 12	3,071 (40.6%)	430 (39.8%)	3,501 (40.5%)	−0.016
	> 12	3,651 (48.3%)	535 (49.5%)	4,186 (48.4%)	0.025
	other	67 (0.9%)	11 (1.0%)	78 (0.9%)	0.014
Equivalent income (million yen)		2.6 (2.1)	2.5 (1.6)	2.6 (2.0)	−0.054
Living arrangement	Living alone	6,660 (88.1%)	949 (87.9%)	7,609 (88.1%)	−0.007
Employment status	unemployed	7,143 (94.5%)	1,028 (95.2%)	8,171 (94.6%)	0.032
Assets (million yen)	< 1	1,299 (17.2%)	218 (20.2%)	1,517 (17.6%)	0.077
	1 ~ 5	383 (5.1%)	62 (5.7%)	445 (5.2%)	0.03
	5 ~ 10	498 (6.6%)	72 (6.7%)	570 (6.6%)	0.003
	10 ~ 50	4,000 (52.9%)	542 (50.2%)	4,542 (52.6%)	−0.055
	> 50	1,380 (18.3%)	186 (17.2%)	1,566 (18.1%)	−0.027
Activities of Daily Living (ADL)	Not need care/assistance	5,532 (73.2%)	801 (74.2%)	6,333 (73.3%)	0.023
	Need but not have care/assistance	956 (12.6%)	127 (11.8%)	1,083 (12.5%)	−0.027
	Need and have care/assistance	1,072 (14.2%)	152 (14.1%)	1,224 (14.2%)	−0.003
Body Mass Index (BMI)		21.4 (3.1)	21.5 (3.0)	21.4 (3.1)	0.009
Self-rated health	Good	6,084 (80.5%)	885 (81.9%)	6,969 (80.7%)	0.038
Diseases in treatment	Have	6,673 (88.3%)	961 (89.0%)	7,634 (88.4%)	0.022
Degree of need for care	Independent	4,715 (62.4%)	671 (62.1%)	5,386 (62.3%)	−0.005

^a^ SHOP: Serviced Housing for Older People

**Table 3:** Comparison of health and well-being between the SHOPa group and the control group after propensity score matching

		Control group	SHOP group	Total	Test
		*n* = 7,560 (87.5%)	*n* = 1,080 (12.5%)	*n* = 8,640 (100.0%)	
Happiness and Life Satisfaction	Happiness	7.1 (1.9)	**7.6 (1.6)**	7.2 (1.8)	**< 0.001**
	Life satisfaction	6.9 (2.0)	**7.2 (1.8)**	6.9 (2.0)	**< 0.001**
Mental and Physical Health	Physical health	6.3 (2.0)	**6.6 (2.0)**	6.4 (2.0)	**< 0.001**
	Mental health	7.0 (2.0)	7.1 (1.9)	7.0 (2.0)	0.618
Meaning and Purpose	Life worthwhile	6.6 (2.2)	6.6 (2.1)	6.6 (2.2)	0.149
	Ikigai	6.7 (2.3)	6.6 (2.2)	6.7 (2.3)	0.060
	Life’s purpose	6.7 (2.3)	6.6 (2.2)	6.7 (2.3)	0.583
Close Social Relationships	Contentment with friendships and relationships	6.6 (2.3)	6.6 (2.1)	6.6 (2.3)	0.349
	Relationship satisfaction	6.9 (2.3)	6.9 (2.1)	6.9 (2.2)	0.324

^a^SHOP: Serviced Housing for Older People

In the sensitivity analysis, after achieving covariate balance in each iteration (Supplementary Tables 1–9), individual comparisons were made for each health and well-being item (Supplementary Table 10). The results showed no significant differences in life satisfaction or physical health. However, mental health, life worthwhile, and ikigai were reported to be worse among the SHOP group than the control group.

Table 4 compares factors associating health and well-being between the SHOP and control groups after PSM, with higher percentages and *p* < 0.05 shown in bold. In the SHOP group, 71.2% of participants laughed almost every day compared to 64.2% in the control group (*p* < 0.001). Additionally, 67.7% of the SHOP group went out at least five times a week, compared to 36.9% in the control group (*p* < 0.001). Furthermore, 34.2% of the SHOP group participated in preventive care activities at least once a month, compared to 22.3% in the control group (*p* < 0.001). Moreover, the SHOP group was over three times more likely to meet with friends at least four times a week compared to the control group (34.4% vs. 10.3%, *p* < 0.001). The SHOP group also reported receiving greater emotional support and more opportunities to eat with others at least once a week. However, no significant differences in depression levels were observed between the two groups.

**Table 4:** Comparison of factors that May support health and well-being between the control group and the SHOPa group after propensity score matching

	Control group	SHOP group	Total	Test
	*n* = 7,560 (87.5%)	*n* = 1,080 (12.5%)	*n* = 8,640 (100.0%)	
Laugh almost every day	4,850 (64.2%)	**769 (71.2%)**	5,619 (65.0%)	**< 0.001**
Go out at least 5 times a week	2,786 (36.9%)	**731 (67.7%)**	3,517 (40.7%)	**< 0.001**
Depression (mean score)	3.9 (3.3)	3.8 (3.2)	3.9 (3.2)	0.309
Have hobbies	6,532 (86.4%)	944 (87.4%)	7,476 (86.5%)	0.365
Participate in preventive care activities at least once a month	1,683 (22.3%)	**369 (34.2%)**	2,052 (23.8%)	**< 0.001**
Meet with friends at least 4 times a week	779 (10.3%)	**372 (34.4%)**	1,151 (13.3%)	**< 0.001**
Receive emotional support	6,930 (91.7%)	**1**,**019 (94.4%)**	7,949 (92.0%)	**0.002**
Eat with others at least once a week	1,967 (26.0%)	**418 (38.7%)**	2,385 (27.6%)	**< 0.001**

^a^SHOP: Serviced Housing for Older People

## Discussion

This study compared the health and well-being of residents in SHOP named “Grandmast” with a control group of community-dwelling older adults. The results indicated that the SHOP group reported higher levels of happiness, life satisfaction, and physical health. However, there was no significant difference in mental health, life meaning and purpose, or close relationships compared to the control group.

Participants in the SHOP group reported higher levels of happiness and life satisfaction compared to the control group. This could be attributed to the environment of SHOP, which includes communal spaces and organized activities that promote social participation and interaction [6, 35]. By facilitating interactions among residents, their families, and friends, SHOP may help reduce social isolation, which can have a negative impact on happiness and life satisfaction [28, 30]. Communal dining spaces in SHOP may also encourage eating with others, which is linked to emotional support and increased happiness [31]. Additionally, engaging in conversations with friends and participating in group exercise classes may lead to more laughter among residents [36], ultimately enhancing happiness and life satisfaction [25]. Although this study did not extensively explore the relationship between factors such as laughter, social outings, participating in community gathering places, meeting with friends, receiving emotional support, and eating with others and the health and well-being of SHOP residents, it was observed that the SHOP group exhibited better outcomes in these aspects compared to the control group.

The SHOP group exhibited higher levels of physical health compared to the control group, possibly due to increased social participation [37], including outings and participation in physical exercise. In this study, the SHOP group demonstrated a higher likelihood of outings at least five times a week compared to the control group (67.7% vs. 36.9%, *p* < 0.001) and attending preventive care activities like professionally guided exercise classes (34.2% vs. 22.3%, *p* < 0.001). These activities promote increased physical activity, essential for maintaining health and preventing functional decline [38]. Previous studies have highlighted the impact of transportation on social participation [39]. Therefore, SHOP in urban areas with convenient transportation and proximity to train stations is more likely to facilitate residents’ engagement in social activities. The concierge desk at SHOP offers information on outdoor locations, activity venues, and exercise opportunities, thereby encouraging participation in community gathering places. Additionally, SHOP regularly organizes professionally guided exercise classes, motivating residents to partake in physical activities, ultimately contributing to enhanced physical health among residents [40]. However, in this study, no significant differences were observed in mental health, life meaning and purpose, or close social relationships between the SHOP and control groups.

Previous research has indicated that moving to senior housing can result in feelings of depression or loneliness due to the challenges of adapting to a new environment and limited social interactions [15, 41]. However, the current study found that SHOP residents did not exhibit low mental health levels, possibly due to the availability of communal spaces for social interactions and support services from the concierge desk, which could help reduce psychological stress and enhance communication among residents [42].

While relocating to a SHOP may disrupt existing social networks, including relationships with family, friends, and neighbors [16, 29], some studies have shown that residents maintain close connections through face-to-face interactions, phone calls, or online communication [17]. In this study, close social relationships did not differ significantly between the two groups, with the SHOP group more likely to meet with friends at least four times a week compared to the control group (34.4% vs. 13.3%). This suggests that relocating to a SHOP may not significantly disrupt close social relationships, possibly due to the short relocation distance [29], or the opportunity to form new social connections within the community [17]. Litwak and Longino [43] classified relocation in old age into three patterns: first at retirement, second due to moderate forms of disability, and third in response to major forms of chronic disability. In our study, most residents relocated proactively, often citing reasons such as “preparation for the future” or “the desire for independent living with peace of mind.” Given that all included SHOP facilities in the study were designed for older adults who can live independently and do not offer medical or caregiving services, most residents potentially align with the first or second relocation pattern. These patterns are typically voluntary moves, rather than crisis-driven relocations, which may help explain the observed maintenance of mental health after relocation. As residents spend more time in the SHOP and engage in communal activities, such as festival celebrations, their social relationships may strengthen, potentially improving mental health.

Finally, it is important to consider the results of sensitivity analysis. The SHOP group consistently showed higher levels of happiness compared to the control group. However, there were no significant differences in life satisfaction and physical health. Conversely, the SHOP group exhibited lower levels of mental health, life purpose, and ikigai. This suggests a correlation between health and well-being items [21], such as meaning in life and close relationships, which are linked to life satisfaction [44]. It is essential to be cautious of over adjustment if some items act as mediators. Additionally, some individuals may relocate to senior housing due to worsening health status [42]. Therefore, the lower mental health, life purpose, and ikigai levels in the SHOP group may not solely be attributed to their current status but could be influenced by their pre-existing levels before moving into SHOP. Additionally, happiness, as evaluated in this study, represents a transient emotion, whereas mental health, life purpose, and ikigai require longer periods to change. Further longitudinal studies are needed to track changes in these aspects over time.

The primary strength of this study is its novelty in providing evidence from Japan comparing the health and well-being of SHOP residents with that of community-dwelling older adults using PSM. This research not only underscores the potential benefits of SHOP for older adults but also examines social participation and interaction aspects that may contribute to differences in health and well-being. The findings offer valuable insights for informing the future design and service provision of senior housing facilities.

However, several limitations should be acknowledged. First, the SHOP in this study targets individuals capable of independent living, whereas some other facilities also offer caregiving services. Most residents are in good health and financially stable, indicating that the findings may not be generalizable to SHOPs that serve residents with more extensive support needs. Future research should include a broader range of SHOP settings to enhance the generalizability of the results. Second, this cross-sectional study could not establish causal relationships between SHOP residency and health and well-being. Longitudinal studies are needed to determine the directionality of these associations and elucidate causal relationships. Third, unmeasured confounders were not accounted for, potentially leading to inadequate matching between the two groups. In particular, variables related to family structure or support—such as coresidence with children, caregiving responsibilities, and proximity to relatives—were not available in the dataset. These factors may influence the decision to enter senior housing and subsequent health and well-being outcomes. Future research should incorporate such contextual variables to improve model accuracy. Furthermore, differences in housing tenure (e.g., ownership vs. rental) may reflect wide disparities in asset composition, housing security, and residential motivations, which were not adjusted for in this analysis. Fourth, the study did not consider participants’ status before moving into the SHOP, such as transitioning from living with others to living alone, which could impact their health and well-being post-relocation. Fifth, the study did not address the physical environment, including the housing interior or geographical location of the residences. Future research should explore these factors to provide a more comprehensive understanding. Sixth, we did not account for potential clustering at the facility or regional level. Although facility-level identifiers were available for SHOP participants and area codes for control participants, the datasets were drawn from separate survey frames and covered only partially overlapping regions. Because of the lack of a consistent hierarchical structure, especially within the control group, multilevel modeling was not feasible. Future studies employing nested designs and unified sampling frameworks could better address region- or facility-level variation using mixed-effects models. Seventh, although both groups were restricted to ordinance-designated cities to enhance comparability, the specific municipalities did not overlap completely. This geographic mismatch may affect comparability. Future studies should consider more precise matching of facility and control areas to improve regional comparability.

## Conclusion

Compared to community-dwelling older adults matched using PSM, SHOP residents demonstrated higher levels of happiness, life satisfaction, and better physical health. No significant differences were observed in mental health, life meaning and purpose, or close social relationships between SHOP residents and those living in the community. SHOP residents reported higher levels of factors that may support health and well-being, such as laughing, going out, participating in preventive care activities, meeting with friends, receiving emotional support, and eating with others. These findings provide valuable insights into the potential benefits of residing in SHOP for older adults’ health and well-being and may contribute to the development of housing models aimed at enhancing well-being in aging populations.

## Electronic Supplementary Material

Below is the link to the electronic supplementary material.

Supplementary Material 1

## Data Availability

The datasets of the Japan Gerontological Evaluation Study are available from the corresponding author upon reasonable request. All enquiries should be addressed to the data management committee via e-mail: dataadmin.ml@jages.net. However, the data collected specifically for this study by Sekisui House Real Estate Tokyo, Ltd, cannot be made publicly available since the participants in this study did not provide consent for their data to be shared publicly.

## Declarations

This study used data from the Japan Gerontological Evaluation Study (JAGES) and Sekisui House Real Estate Tokyo, Ltd. We thank the study participants and the research assistants who collected and managed the data.
