# Supplementary material for: Health and well-being comparison between residents of serviced housing for older people and community-dwelling older adults in japan: a propensity score matching analysis
Source: Soc Psychiatry Psychiatr Epidemiol. 2025 Jun 23;61(6):1081–90. doi: 10.1007/s00127-025-02947-8 (PMC13226352; doi:10.1007/s00127-025-02947-8)
Supplement: Supplementary file 1 — Supplementary Material 1 [file 127_2025_2947_MOESM1_ESM.docx]

**Supplementary Fig 1** Flowchart of participant inclusion


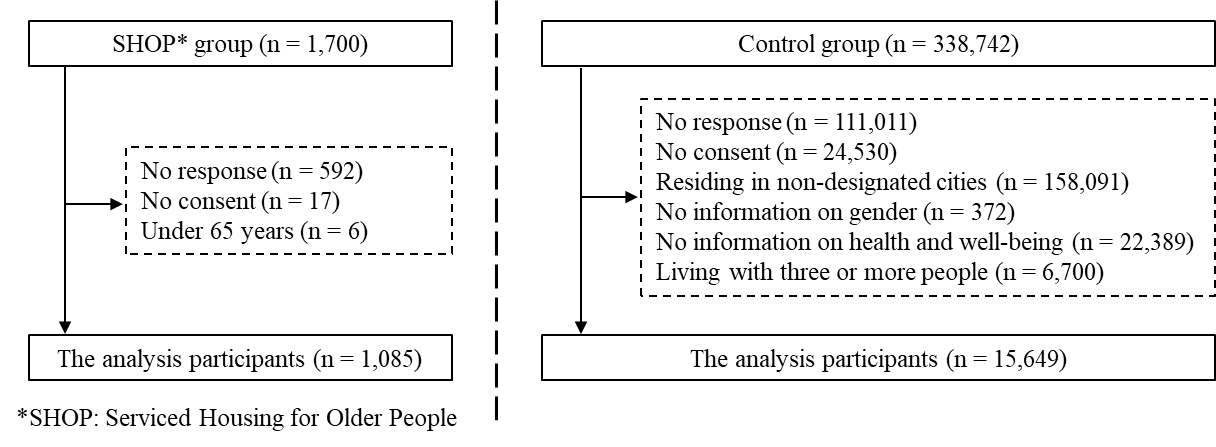


**Supplementary Fig 2** Distribution of propensity scores between the SHOP group and the control group. SHOP: Serviced Housing for Older People


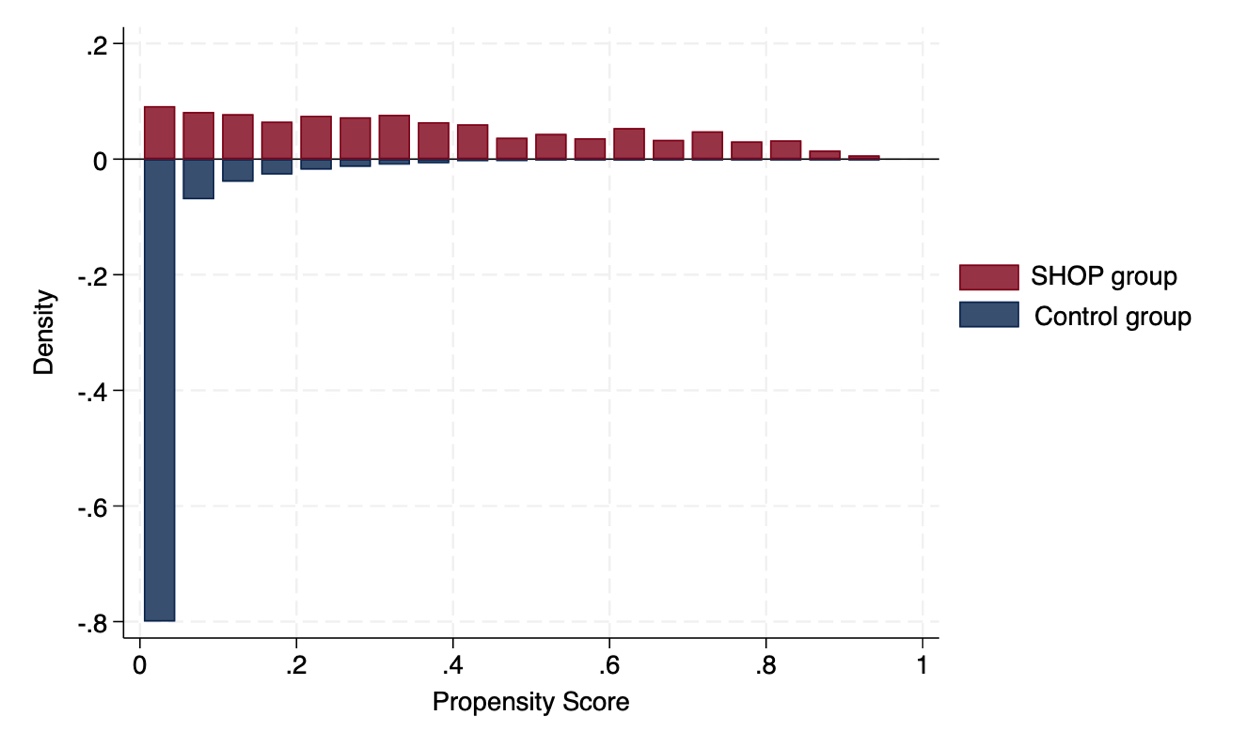


| **Supplementary Table 1** Characteristics of the control and the SHOP^a^ group after propensity score matching for the analysis of happiness | | | | | |
| --- | --- | --- | --- | --- | --- |
|  |  | Control group | SHOP group | Total | SD |
|  |  | n=7,560 (87.5%) | n=1,080 (12.5%) | n=8,640 (100.0%) |  |
| Sex | Female | 5,060 (66.9%) | 750 (69.4%) | 5,810 (67.2%) | 0.054 |
| Age |  | 84.1 (6.6) | 83.9 (6.5) | 84.1 (6.6) | -0.038 |
| Education (years) | <10 | 769 (10.2%) | 104 (9.6%) | 873 (10.1%) | -0.018 |
|  | 10~12 | 3,040 (40.2%) | 430 (39.8%) | 3,470 (40.2%) | -0.008 |
|  | >12 | 3,660 (48.4%) | 535 (49.5%) | 4,195 (48.6%) | 0.022 |
|  | Other | 91 (1.2%) | 11 (1.0%) | 102 (1.2%) | -0.018 |
| Equivalent income (million yen) |  | 2.6 (2.1) | 2.5 (1.5) | 2.6 (2.0) | -0.075 |
| Living arrangement | Living alone | 6,646 (87.9%) | 949 (87.9%) | 7,595 (87.9%) | -0.001 |
| Employment status | Unemployed | 7,102 (93.9%) | 1,028 (95.2%) | 8,130 (94.1%) | 0.055 |
| Assets (million yen) | <1 | 1,257 (16.6%) | 218 (20.2%) | 1,475 (17.1%) | 0.092 |
|  | 1~5 | 361 (4.8%) | 62 (5.7%) | 423 (4.9%) | 0.043 |
|  | 5~10 | 538 (7.1%) | 72 (6.7%) | 610 (7.1%) | -0.018 |
|  | 10~50 | 3,995 (52.8%) | 542 (50.2%) | 4,537 (52.5%) | -0.053 |
|  | >50 | 1,409 (18.6%) | 186 (17.2%) | 1,595 (18.5%) | -0.037 |
| Activities of daily living (ADL) | Not need care/assistance | 5,645 (74.7%) | 801 (74.2%) | 6,446 (74.6%) | -0.012 |
|  | Need but not have care/assistance | 923 (12.2%) | 127 (11.8%) | 1,050 (12.2%) | -0.014 |
|  | Need and have care/assistance | 992 (13.1%) | 152 (14.1%) | 1,144 (13.2%) | 0.028 |
| Body mass index (BMI) |  | 21.4 (3.1) | 21.5 (3.0) | 21.4 (3.1) | 0.012 |
| Self-rated health | Good | 6,180 (81.7%) | 885 (81.9%) | 7,065 (81.8%) | 0.012 |
| Diseases in treatment | Have | 6,668 (88.2%) | 961 (89.0%) | 7,629 (88.3%) | 0.025 |
| Degree of need for care | Independent | 4,666 (61.7%) | 671 (62.1%) | 5,337 (61.8%) | 0.025 |
| Life satisfaction | 0 ~ 10points | 7.2 (1.9) | 7.2 (1.8) | 7.2 (1.9) | 0.005 |
| Physical health | 0 ~ 10points | 6.6 (2.0) | 6.6 (2.0) | 6.6 (2.0) | 0.015 |
| Mental health | 0 ~ 10points | 7.1 (2.0) | 7.1 (1.9) | 7.1 (2.0) | 0.004 |
| Life worthwhile | 0 ~ 10points | 6.6 (2.2) | 6.6 (2.1) | 6.6 (2.2) | -0.01 |
| Ikigai | 0 ~ 10points | 6.7 (2.4) | 6.6 (2.2) | 6.7 (2.4) | -0.02 |
| Life’s purpose | 0 ~ 10points | 6.7 (2.3) | 6.6 (2.2) | 6.7 (2.3) | -0.02 |
| Contentment with friendships and relationships | 0 ~ 10points | 6.7 (2.3) | 6.6 (2.1) | 6.7 (2.3) | -0.021 |
| Relationship satisfaction | 0 ~ 10points | 7.0 (2.3) | 6.9 (2.1) | 7.0 (2.2) | -0.026 |
| ^a^ SHOP: Serviced Housing for Older People | | | | | |

| **Supplementary Table 2** Characteristics of the control and the SHOP^a^ group after propensity score matching for the analysis of life satisfaction | | | | | | | | | | | |
| --- | --- | --- | --- | --- | --- | --- | --- | --- | --- | --- | --- |
| Life satisfaction | |  | | Control group | | SHOP group | | Total | | | SD |
|  | |  | | n=7,560 (87.5%) | | n=1,080 (12.5%) | | n=8,640 (100.0%) | | |  |
| Sex | | Female | | 5,034 (66.6%) | | 750 (69.4%) | | 5,784 (66.9%) | | | 0.061 |
| Age | |  | | 84.1 (6.6) | | 83.9 (6.5) | | 84.1 (6.6) | | | -0.039 |
| Education (years) | | <10 | | 799 (10.6%) | | 104 (9.6%) | | 903 (10.5%) | | | -0.031 |
|  |  | 10~12 | | 3,001 (39.7%) | | 430 (39.8%) | | 3,431 (39.7%) | | | 0.002 |
|  |  | >12 | | 3,694 (48.9%) | | 535 (49.5%) | | 4,229 (48.9%) | | | 0.013 |
|  |  | Other | | 66 (0.9%) | | 11 (1.0%) | | 77 (0.9%) | | | 0.015 |
| Equivalent income (million yen) | |  | | 2.6 (2.1) | | 2.5 (1.5) | | 2.6 (2.0) | | | -0.074 |
| Living arrangement | | Living alone | | 7,098 (93.9%) | | 1,028 (95.2%) | | 8,126 (94.1%) | | | 0.004 |
| Employment status | | Unemployed | | 7,098 (93.9%) | | 1,028 (95.2%) | | 8,126 (94.1%) | | | 0.057 |
| Assets (million yen) | | <1 | | 1,301 (17.2%) | | 218 (20.2%) | | 1,519 (17.6%) | | | 0.076 |
|  |  | 1~5 | | 345 (4.6%) | | 62 (5.7%) | | 407 (4.7%) | | | 0.053 |
|  |  | 5~10 | | 509 (6.7%) | | 72 (6.7%) | | 581 (6.7%) | | | -0.003 |
|  |  | 10~50 | | 3,944 (52.2%) | | 542 (50.2%) | | 4,486 (51.9%) | | | -0.04 |
|  |  | >50 | | 1,461 (19.3%) | | 186 (17.2%) | | 1,647 (19.1%) | | | -0.054 |
| Activities of daily living (ADL) | | Not need care/assistance | | 5,606 (74.2%) | | 801 (74.2%) | | 6,407 (74.2%) | | | 0 |
|  |  | Need but not have care/assistance | | 951 (12.6%) | | 127 (11.8%) | | 1,078 (12.5%) | | | -0.025 |
|  |  | Need and have care/assistance | | 1,003 (13.3%) | | 152 (14.1%) | | 1,155 (13.4%) | | | 0.023 |
| Body mass index (BMI) | |  | | 21.4 (3.1) | | 21.5 (3.0) | | 21.4 (3.1) | | | 0.013 |
| Self-rated health | | Good | | 6,106 (80.8%) | | 885 (81.9%) | | 6,991 (80.9%) | | | 0.03 |
| Diseases in treatment | | Have | | 6,717 (88.8%) | | 961 (89.0%) | | 7,678 (88.9%) | | | 0.004 |
| Degree of need for care | | Independent | | 4,719 (62.4%) | | 671 (62.1%) | | 5,390 (62.4%) | | | -0.006 |
| Happiness | | 0 ~ 10points | | 7.7 (1.8) | | 7.6 (1.6) | | 7.7 (1.7) | | | -0.014 |
| Physical health | | 0 ~ 10points | | 6.5 (2.0) | | 6.6 (2.0) | | 6.5 (2.0) | | | 0.042 |
| Mental health | | 0 ~ 10points | | 7.1 (2.1) | | 7.1 (1.9) | | 7.1 (2.1) | | | 0.004 |
| Life worthwhile | | 0 ~ 10points | | 6.6 (2.3) | | 6.6 (2.1) | | 6.6 (2.3) | | | -0.019 |
| Ikigai | | 0 ~ 10points | | 6.7 (2.4) | | 6.6 (2.2) | | 6.7 (2.4) | | | -0.018 |
| Life’s purpose | | 0 ~ 10points | | 6.7 (2.4) | | 6.6 (2.2) | | 6.7 (2.4) | | | -0.011 |
| Contentment with friendships and relationships | | 0 ~ 10points | | 6.7 (2.4) | | 6.6 (2.1) | | 6.7 (2.3) | | | -0.01 |
| Relationship satisfaction | | 0 ~ 10points | | 7.0 (2.3) | | 6.9 (2.1) | | 7.0 (2.3) | | | -0.014 |
| ^a^ SHOP: Serviced Housing for Older People | | | | | | | | | | | |
|  |  | |  | |  | |  | |  |  |  |

| **Supplementary Table 3** Characteristics of the control and the SHOP^a^ group after propensity score matching for the analysis of physical health | | | | | | | | | | | |
| --- | --- | --- | --- | --- | --- | --- | --- | --- | --- | --- | --- |
| Physical health | |  | | Control group | | SHOP group | | Total | | | SD |
|  | |  | | n=7,560 (87.5%) | | n=1,080 (12.5%) | | n=8,640 (100.0%) | | |  |
| Sex | | Female | | 5,071 (67.1%) | | 750 (69.4%) | | 5,821 (67.4%) | | | 0.051 |
| Age | |  | | 84.1 (6.5) | | 83.9 (6.5) | | 84.1 (6.5) | | | -0.033 |
| Education (years) | | <10 | | 808 (10.7%) | | 104 (9.6%) | | 912 (10.6%) | | | -0.035 |
|  |  | 10~12 | | 3,002 (39.7%) | | 430 (39.8%) | | 3,432 (39.7%) | | | 0.002 |
|  |  | >12 | | 3,673 (48.6%) | | 535 (49.5%) | | 4,208 (48.7%) | | | 0.019 |
|  |  | Other | | 77 (1.0%) | | 11 (1.0%) | | 88 (1.0%) | | | 0.000 |
| Equivalent income (million yen) | |  | | 2.6 (2.1) | | 2.5 (1.5) | | 2.6 (2.1) | | | -0.074 |
| Living arrangement | | Living alone | | 6,642 (87.9%) | | 949 (87.9%) | | 7,591 (87.9%) | | | 0.000 |
| Employment status | | Unemployed | | 7,128 (94.3%) | | 1,028 (95.2%) | | 8,156 (94.4%) | | | 0.040 |
| Assets (million yen) | | <1 | | 1,323 (17.5%) | | 218 (20.2%) | | 1,541 (17.8%) | | | 0.069 |
|  |  | 1~5 | | 408 (5.4%) | | 62 (5.7%) | | 470 (5.4%) | | | 0.015 |
|  |  | 5~10 | | 503 (6.7%) | | 72 (6.7%) | | 575 (6.7%) | | | 0.001 |
|  |  | 10~50 | | 3,953 (52.3%) | | 542 (50.2%) | | 4,495 (52.0%) | | | -0.042 |
|  |  | >50 | | 1,373 (18.2%) | | 186 (17.2%) | | 1,559 (18.0%) | | | -0.025 |
| Activities of daily living (ADL) | | Not need care/assistance | | 5,613 (74.2%) | | 801 (74.2%) | | 6,414 (74.2%) | | | -0.002 |
|  |  | Need but not have care/assistance | | 955 (12.6%) | | 127 (11.8%) | | 1,082 (12.5%) | | | -0.027 |
|  |  | Need and have care/assistance | | 992 (13.1%) | | 152 (14.1%) | | 1,144 (13.2%) | | | 0.028 |
| Body mass index (BMI) | |  | | 21.5 (3.1) | | 21.5 (3.0) | | 21.5 (3.1) | | | -0.021 |
| Self-rated health | | Good | | 6,108 (80.8%) | | 885 (81.9%) | | 6,993 (80.9%) | | | 0.030 |
| Diseases in treatment | | Have | | 6,706 (88.7%) | | 961 (89.0%) | | 7,667 (88.7%) | | | 0.009 |
| Degree of need for care | | Independent | | 4,745 (62.8%) | | 671 (62.1%) | | 5,416 (62.7%) | | | -0.013 |
| Happiness | | 0 ~ 10points | | 7.7 (1.7) | | 7.6 (1.6) | | 7.7 (1.7) | | | -0.033 |
| Life satisfaction | | 0 ~ 10points | | 7.3 (1.9) | | 7.2 (1.8) | | 7.3 (1.9) | | | -0.039 |
| Mental health | | 0 ~ 10points | | 7.1 (2.1) | | 7.1 (1.9) | | 7.1 (2.0) | | | -0.010 |
| Life worthwhile | | 0 ~ 10points | | 6.6 (2.3) | | 6.6 (2.1) | | 6.6 (2.2) | | | -0.032 |
| Ikigai | | 0 ~ 10points | | 6.7 (2.4) | | 6.6 (2.2) | | 6.7 (2.4) | | | -0.027 |
| Life’s purpose | | 0 ~ 10points | | 6.7 (2.3) | | 6.6 (2.2) | | 6.7 (2.3) | | | -0.031 |
| Contentment with friendships and relationships | | 0 ~ 10points | | 6.7 (2.3) | | 6.6 (2.1) | | 6.7 (2.3) | | | -0.036 |
| Relationship satisfaction | | 0 ~ 10points | | 7.0 (2.3) | | 6.9 (2.1) | | 7.0 (2.2) | | | -0.042 |
| ^a^ SHOP: Serviced Housing for Older People | | | | | | | | | | | |
|  |  | |  | |  | |  | |  |  |  |

| **Supplementary Table 4** Characteristics of the control and the SHOP^a^ group after propensity score matching for the analysis of mental health | | | | | |
| --- | --- | --- | --- | --- | --- |
|  |  | Control group | SHOP group | Total | SD |
|  |  | n=7,560 (87.5%) | n=1,080 (12.5%) | n=8,640 (100.0%) |  |
| Sex | Female | 5,108 (67.6%) | 750 (69.4%) | 5,858 (67.8%) | 0.04 |
| Age |  | 84.1 (6.6) | 83.9 (6.5) | 84.0 (6.6) | -0.027 |
| Education (years) | <10 | 826 (10.9%) | 104 (9.6%) | 930 (10.8%) | -0.043 |
|  | 10~12 | 3,102 (41.0%) | 430 (39.8%) | 3,532 (40.9%) | -0.025 |
|  | >12 | 3,574 (47.3%) | 535 (49.5%) | 4,109 (47.6%) | 0.045 |
|  | Other | 58 (0.8%) | 11 (1.0%) | 69 (0.8%) | 0.027 |
| Equivalent income (million yen) |  | 2.6 (2.1) | 2.5 (1.5) | 2.6 (2.0) | -0.066 |
| Living arrangement | Living alone | 6,653 (88.0%) | 949 (87.9%) | 7,602 (88.0%) | -0.004 |
| Employment status | Unemployed | 7,140 (94.4%) | 1,028 (95.2%) | 8,168 (94.5%) | 0.033 |
| Assets (million yen) | <1 | 1,393 (18.4%) | 218 (20.2%) | 1,611 (18.6%) | 0.045 |
|  | 1~5 | 367 (4.9%) | 62 (5.7%) | 429 (5.0%) | 0.04 |
|  | 5~10 | 535 (7.1%) | 72 (6.7%) | 607 (7.0%) | -0.016 |
|  | 10~50 | 3,945 (52.2%) | 542 (50.2%) | 4,487 (51.9%) | -0.04 |
|  | >50 | 1,320 (17.5%) | 186 (17.2%) | 1,506 (17.4%) | -0.006 |
| Activities of daily living (ADL) | Not need care/assistance | 5,495 (72.7%) | 801 (74.2%) | 6,296 (72.9%) | 0.034 |
|  | Need but not have care/assistance | 1,045 (13.8%) | 127 (11.8%) | 1,172 (13.6%) | -0.062 |
|  | Need and have care/assistance | 1,020 (13.5%) | 152 (14.1%) | 1,172 (13.6%) | 0.017 |
| Body mass index (BMI) |  | 21.5 (3.1) | 21.5 (3.0) | 21.5 (3.1) | 0.004 |
| Self-rated health | Good | 6,031 (79.8%) | 885 (81.9%) | 6,916 (80.0%) | 0.055 |
| Diseases in treatment | Have | 6,654 (88.0%) | 961 (89.0%) | 7,615 (88.1%) | 0.03 |
| Degree of need for care | Independent | 4,640 (61.4%) | 671 (62.1%) | 5,311 (61.5%) | 0.016 |
| Happiness | 0 ~ 10points | 7.7 (1.8) | 7.6 (1.6) | 7.7 (1.7) | -0.005 |
| Life satisfaction | 0 ~ 10points | 7.3 (2.0) | 7.2 (1.8) | 7.3 (2.0) | -0.014 |
| Physical health | 0 ~ 10points | 6.6 (2.0) | 6.6 (2.0) | 6.6 (2.0) | 0.026 |
| Life worthwhile | 0 ~ 10points | 6.6 (2.3) | 6.6 (2.1) | 6.6 (2.3) | -0.015 |
| Ikigai | 0 ~ 10points | 6.7 (2.5) | 6.6 (2.2) | 6.7 (2.4) | -0.014 |
| Life’s purpose | 0 ~ 10points | 6.7 (2.4) | 6.6 (2.2) | 6.6 (2.4) | 0 |
| Contentment with friendships and relationships | 0 ~ 10points | 6.7 (2.4) | 6.6 (2.1) | 6.7 (2.3) | -0.008 |
| Relationship satisfaction | 0 ~ 10points | 7.0 (2.3) | 6.9 (2.1) | 7.0 (2.3) | -0.024 |
| ^a^ SHOP: Serviced Housing for Older People | | | | | |

| **Supplementary Table 5** Characteristics of the control and the SHOP^a^ group after propensity score matching for the analysis of life worthwhile | | | | | |
| --- | --- | --- | --- | --- | --- |
|  |  | Control group | SHOP group | Total | SD |
|  |  | n=7,560 (87.5%) | n=1,080 (12.5%) | n=8,640 (100.0%) |  |
| Sex | Female | 5,077 (67.2%) | 750 (69.4%) | 5,827 (67.4%) | 0.049 |
| Age |  | 84.1 (6.5) | 83.9 (6.5) | 84.1 (6.5) | -0.034 |
| Education (years) | <10 | 839 (11.1%) | 104 (9.6%) | 943 (10.9%) | -0.048 |
|  | 10~12 | 3,088 (40.8%) | 430 (39.8%) | 3,518 (40.7%) | -0.021 |
|  | >12 | 3,573 (47.3%) | 535 (49.5%) | 4,108 (47.5%) | 0.046 |
|  | Other | 60 (0.8%) | 11 (1.0%) | 71 (0.8%) | 0.024 |
| Equivalent income (million yen) |  | 2.6 (2.1) | 2.5 (1.5) | 2.6 (2.0) | -0.058 |
| Living arrangement | Living alone | 6,611 (87.4%) | 949 (87.9%) | 7,560 (87.5%) | 0.013 |
| Employment status | Unemployed | 7,161 (94.7%) | 1,028 (95.2%) | 8,189 (94.8%) | 0.021 |
| Assets (million yen) | <1 | 1,332 (17.6%) | 218 (20.2%) | 1,550 (17.9%) | 0.066 |
|  | 1~5 | 364 (4.8%) | 62 (5.7%) | 426 (4.9%) | 0.041 |
|  | 5~10 | 540 (7.1%) | 72 (6.7%) | 612 (7.1%) | -0.019 |
|  | 10~50 | 3,958 (52.4%) | 542 (50.2%) | 4,500 (52.1%) | -0.043 |
|  | >50 | 1,366 (18.1%) | 186 (17.2%) | 1,552 (18.0%) | -0.022 |
| Activities of daily living (ADL) | Not need care/assistance | 5,550 (73.4%) | 801 (74.2%) | 6,351 (73.5%) | 0.017 |
|  | Need but not have care/assistance | 1,025 (13.6%) | 127 (11.8%) | 1,152 (13.3%) | -0.054 |
|  | Need and have care/assistance | 985 (13.0%) | 152 (14.1%) | 1,137 (13.2%) | 0.031 |
| Body mass index (BMI) |  | 21.5 (3.1) | 21.5 (3.0) | 21.5 (3.1) | 0.005 |
| Self-rated health | Good | 6,118 (80.9%) | 885 (81.9%) | 7,003 (81.1%) | 0.026 |
| Diseases in treatment | Have | 6,667 (88.2%) | 961 (89.0%) | 7,628 (88.3%) | 0.025 |
| Degree of need for care | Independent | 4,683 (61.9%) | 671 (62.1%) | 5,354 (62.0%) | 0.004 |
| Happiness | 0 ~ 10points | 7.7 (1.8) | 7.6 (1.6) | 7.7 (1.7) | -0.034 |
| Life satisfaction | 0 ~ 10points | 7.3 (1.9) | 7.2 (1.8) | 7.3 (1.9) | -0.042 |
| Physical health | 0 ~ 10points | 6.6 (1.9) | 6.6 (2.0) | 6.6 (2.0) | 0.001 |
| Mental health | 0 ~ 10points | 7.1 (2.0) | 7.1 (1.9) | 7.1 (2.0) | -0.019 |
| Ikigai | 0 ~ 10points | 6.7 (2.4) | 6.6 (2.2) | 6.7 (2.4) | -0.013 |
| Life’s purpose | 0 ~ 10points | 6.7 (2.3) | 6.6 (2.2) | 6.7 (2.3) | -0.041 |
| Contentment with friendships and relationships | 0 ~ 10points | 6.7 (2.3) | 6.6 (2.1) | 6.7 (2.2) | -0.03 |
| Relationship satisfaction | 0 ~ 10points | 7.0 (2.3) | 6.9 (2.1) | 7.0 (2.3) | -0.027 |
| ^a^ SHOP: Serviced Housing for Older People | | | | | |

| **Supplementary Table 6** Characteristics of the control and the SHOP^a^ group after propensity score matching for the analysis of ikigai | | | | | |
| --- | --- | --- | --- | --- | --- |
|  |  | Control group | SHOP group | Total | SD |
|  |  | n=7,560 (87.5%) | n=1,080 (12.5%) | n=8,640 (100.0%) |  |
| Sex | Female | 5,104 (67.5%) | 750 (69.4%) | 5,854 (67.8%) | 0.042 |
| Age |  | 84.0 (6.7) | 83.9 (6.5) | 84.0 (6.7) | -0.017 |
| Education (years) | <10 | 831 (11.0%) | 104 (9.6%) | 935 (10.8%) | -0.045 |
|  | 10~12 | 3,071 (40.6%) | 430 (39.8%) | 3,501 (40.5%) | -0.016 |
|  | >12 | 3,592 (47.5%) | 535 (49.5%) | 4,127 (47.8%) | 0.041 |
|  | Other | 66 (0.9%) | 11 (1.0%) | 77 (0.9%) | 0.015 |
| Equivalent income (million yen) | 2.6 (2.2) | 2.5 (1.5) | 2.6 (2.1) | -0.089 |  |
| Living arrangement | Living alone | 6,637 (87.8%) | 949 (87.9%) | 7,586 (87.8%) | 0.002 |
| Employment status | Unemployed | 7,127 (94.3%) | 1,028 (95.2%) | 8,155 (94.4%) | 0.041 |
| Assets (million yen) | <1 | 1,300 (17.2%) | 218 (20.2%) | 1,518 (17.6%) | 0.077 |
|  | 1~5 | 340 (4.5%) | 62 (5.7%) | 402 (4.7%) | 0.056 |
|  | 5~10 | 491 (6.5%) | 72 (6.7%) | 563 (6.5%) | 0.007 |
|  | 10~50 | 4,015 (53.1%) | 542 (50.2%) | 4,557 (52.7%) | -0.059 |
|  | >50 | 1,414 (18.7%) | 186 (17.2%) | 1,600 (18.5%) | -0.039 |
| Activities of daily living (ADL) | Not need care/assistance | 5,532 (73.2%) | 801 (74.2%) | 6,333 (73.3%) | 0.023 |
|  | Need but not have care/assistance | 1,020 (13.5%) | 127 (11.8%) | 1,147 (13.3%) | -0.052 |
|  | Need and have care/assistance | 1,008 (13.3%) | 152 (14.1%) | 1,160 (13.4%) | 0.022 |
| Body mass index (BMI) |  | 21.5 (3.1) | 21.5 (3.0) | 21.5 (3.1) | 0.001 |
| Self-rated health | Good | 6,063 (80.2%) | 885 (81.9%) | 6,948 (80.4%) | 0.045 |
| Diseases in treatment | Have | 6,649 (87.9%) | 961 (89.0%) | 7,610 (88.1%) | 0.032 |
| Degree of need for care | Independent | 4,656 (61.6%) | 671 (62.1%) | 5,327 (61.7%) | 0.011 |
| Happiness | 0 ~ 10points | 7.7 (1.7) | 7.6 (1.6) | 7.7 (1.7) | -0.015 |
| Life satisfaction | 0 ~ 10points | 7.3 (2.0) | 7.2 (1.8) | 7.3 (1.9) | -0.032 |
| Physical health | 0 ~ 10points | 6.5 (2.0) | 6.6 (2.0) | 6.6 (2.0) | 0.036 |
| Mental health | 0 ~ 10points | 7.1 (2.1) | 7.1 (1.9) | 7.1 (2.0) | 0.005 |
| Life worthwhile | 0 ~ 10points | 6.5 (2.3) | 6.6 (2.1) | 6.5 (2.3) | 0.006 |
| Life’s purpose | 0 ~ 10points | 6.7 (2.4) | 6.6 (2.2) | 6.7 (2.4) | -0.008 |
| Contentment with friendships and relationships | 0 ~ 10points | 6.6 (2.4) | 6.6 (2.1) | 6.6 (2.3) | 0.000 |
| Relationship satisfaction | 0 ~ 10points | 6.9 (2.3) | 6.9 (2.1) | 6.9 (2.3) | -0.002 |
| ^a^ SHOP: Serviced Housing for Older People | | | | | |

| **Supplementary Table 7** Characteristics of the control and the SHOP^a^ group after propensity score matching for the analysis of life’s purpose | | | | | |
| --- | --- | --- | --- | --- | --- |
|  |  | Control group | SHOP group | Total | SD |
|  |  | n=7,560 (87.5%) | n=1,080 (12.5%) | n=8,640 (100.0%) |  |
| Sex | Female | 5,134 (67.9%) | 750 (69.4%) | 5,884 (68.1%) | 0.033 |
| Age |  | 84.0 (6.7) | 83.9 (6.5) | 84.0 (6.7) | -0.027 |
| Education (years) | <10 | 753 (10.0%) | 104 (9.6%) | 857 (9.9%) | -0.011 |
|  | 10~12 | 3,118 (41.2%) | 430 (39.8%) | 3,548 (41.1%) | -0.029 |
|  | >12 | 3,618 (47.9%) | 535 (49.5%) | 4,153 (48.1%) | 0.034 |
|  | Other | 71 (0.9%) | 11 (1.0%) | 82 (0.9%) | 0.008 |
| Equivalent income (million yen) | 2.6 (2.1) | 2.5 (1.5) | 2.6 (2.0) | -0.075 |  |
| Living arrangement | Living alone | 6,648 (87.9%) | 949 (87.9%) | 7,597 (87.9%) | -0.002 |
| Employment status | Unemployed | 7,103 (94.0%) | 1,028 (95.2%) | 8,131 (94.1%) | 0.054 |
| Assets (million yen) | <1 | 1,289 (17.1%) | 218 (20.2%) | 1,507 (17.4%) | 0.081 |
|  | 1~5 | 408 (5.4%) | 62 (5.7%) | 470 (5.4%) | 0.015 |
|  | 5~10 | 525 (6.9%) | 72 (6.7%) | 597 (6.9%) | -0.011 |
|  | 10~50 | 3,903 (51.6%) | 542 (50.2%) | 4,445 (51.4%) | -0.029 |
|  | >50 | 1,435 (19.0%) | 186 (17.2%) | 1,621 (18.8%) | -0.046 |
| Activities of daily living (ADL) | Not need care/assistance | 5,513 (72.9%) | 801 (74.2%) | 6,314 (73.1%) | 0.028 |
|  | Need but not have care/assistance | 1,021 (13.5%) | 127 (11.8%) | 1,148 (13.3%) | -0.053 |
|  | Need and have care/assistance | 1,026 (13.6%) | 152 (14.1%) | 1,178 (13.6%) | 0.015 |
| Body mass index (BMI) |  | 21.4 (3.1) | 21.5 (3.0) | 21.4 (3.1) | 0.017 |
| Self-rated health | Good | 6,049 (80.0%) | 885 (81.9%) | 6,934 (80.3%) | 0.049 |
| Diseases in treatment | Have | 6,660 (88.1%) | 961 (89.0%) | 7,621 (88.2%) | 0.028 |
| Degree of need for care | Independent | 4,683 (61.9%) | 671 (62.1%) | 5,354 (62.0%) | 0.004 |
| Happiness | 0 ~ 10points | 7.7 (1.7) | 7.6 (1.6) | 7.7 (1.7) | -0.02 |
| Life satisfaction | 0 ~ 10points | 7.3 (2.0) | 7.2 (1.8) | 7.3 (1.9) | -0.012 |
| Physical health | 0 ~ 10points | 6.5 (1.9) | 6.6 (2.0) | 6.5 (1.9) | 0.04 |
| Mental health | 0 ~ 10points | 7.1 (2.0) | 7.1 (1.9) | 7.1 (2.0) | 0.019 |
| Life worthwhile | 0 ~ 10points | 6.5 (2.3) | 6.6 (2.1) | 6.5 (2.2) | 0.006 |
| Ikigai | 0 ~ 10points | 6.6 (2.4) | 6.6 (2.2) | 6.6 (2.4) | 0.003 |
| Contentment with friendships and relationships | 0 ~ 10points | 6.7 (2.3) | 6.6 (2.1) | 6.7 (2.3) | -0.007 |
| Relationship satisfaction | 0 ~ 10points | 7.0 (2.3) | 6.9 (2.1) | 7.0 (2.3) | -0.019 |
| ^a^ SHOP: Serviced Housing for Older People | | | | | |

| **Supplementary Table 8** Characteristics of the control and the SHOP^a^ group after propensity score matching for the analysis of contentment with friendships and relationships | | | | | |
| --- | --- | --- | --- | --- | --- |
|  |  | Control group | SHOP group | Total | SD |
|  |  | n=7,560 (87.5%) | n=1,080 (12.5%) | n=8,640 (100.0%) |  |
| Sex | Female | 5,084 (67.2%) | 750 (69.4%) | 5,834 (67.5%) | 0.047 |
| Age |  | 84.1 (6.6) | 83.9 (6.5) | 84.1 (6.6) | -0.039 |
| Education (years) | <10 | 812 (10.7%) | 104 (9.6%) | 916 (10.6%) | -0.037 |
|  | 10~12 | 3,108 (41.1%) | 430 (39.8%) | 3,538 (40.9%) | -0.026 |
|  | >12 | 3,562 (47.1%) | 535 (49.5%) | 4,097 (47.4%) | 0.048 |
|  | Other | 78 (1.0%) | 11 (1.0%) | 89 (1.0%) | -0.001 |
| Equivalent income (million yen) |  | 2.6 (2.1) | 2.5 (1.5) | 2.6 (2.1) | -0.068 |
| Living arrangement | Living alone | 6,619 (87.6%) | 949 (87.9%) | 7,568 (87.6%) | 0.01 |
| Employment status | Unemployed | 7,121 (94.2%) | 1,028 (95.2%) | 8,149 (94.3%) | 0.044 |
| Assets (million yen) | <1 | 1,351 (17.9%) | 218 (20.2%) | 1,569 (18.2%) | 0.059 |
|  | 1~5 | 388 (5.1%) | 62 (5.7%) | 450 (5.2%) | 0.027 |
|  | 5~10 | 505 (6.7%) | 72 (6.7%) | 577 (6.7%) | -0.001 |
|  | 10~50 | 3,965 (52.4%) | 542 (50.2%) | 4,507 (52.2%) | -0.045 |
|  | >50 | 1,351 (17.9%) | 186 (17.2%) | 1,537 (17.8%) | -0.017 |
| Activities of daily living (ADL) | Not need care/assistance | 5,548 (73.4%) | 801 (74.2%) | 6,349 (73.5%) | 0.018 |
|  | Need but not have care/assistance | 1,015 (13.4%) | 127 (11.8%) | 1,142 (13.2%) | -0.05 |
|  | Need and have care/assistance | 997 (13.2%) | 152 (14.1%) | 1,149 (13.3%) | 0.026 |
| Body mass index (BMI) |  | 21.4 (3.1) | 21.5 (3.0) | 21.4 (3.1) | 0.012 |
| Self-rated health | Good | 6,113 (80.9%) | 885 (81.9%) | 6,998 (81.0%) | 0.028 |
| Diseases in treatment | Have | 6,615 (87.5%) | 961 (89.0%) | 7,576 (87.7%) | 0.046 |
| Degree of need for care | Independent | 4,680 (61.9%) | 671 (62.1%) | 5,351 (61.9%) | 0.005 |
| Happiness | 0 ~ 10points | 7.7 (1.7) | 7.6 (1.6) | 7.7 (1.7) | -0.027 |
| Life satisfaction | 0 ~ 10points | 7.3 (2.0) | 7.2 (1.8) | 7.3 (1.9) | -0.043 |
| Physical health | 0 ~ 10points | 6.6 (2.0) | 6.6 (2.0) | 6.6 (2.0) | 0.009 |
| Mental health | 0 ~ 10points | 7.1 (2.0) | 7.1 (1.9) | 7.1 (2.0) | -0.005 |
| Life worthwhile | 0 ~ 10points | 6.6 (2.3) | 6.6 (2.1) | 6.6 (2.3) | -0.022 |
| Ikigai | 0 ~ 10points | 6.7 (2.5) | 6.6 (2.2) | 6.7 (2.4) | -0.018 |
| Life’s purpose | 0 ~ 10points | 6.7 (2.4) | 6.6 (2.2) | 6.7 (2.3) | -0.026 |
| Relationship satisfaction | 0 ~ 10points | 7.0 (2.3) | 6.9 (2.1) | 7.0 (2.3) | -0.036 |
| ^a^ SHOP: Serviced Housing for Older People | | | | | |

| **Supplementary Table 9** Characteristics of the control and the SHOP^a^ group after propensity score matching for the analysis of relationship satisfaction | | | | | |
| --- | --- | --- | --- | --- | --- |
|  |  | Control group | SHOP group | Total | SD |
|  |  | n=7,560 (87.5%) | n=1,080 (12.5%) | n=8,640 (100.0%) |  |
| Sex | Female | 5,165 (68.3%) | 750 (69.4%) | 5,915 (68.5%) | 0.024 |
| Age |  | 84.1 (6.6) | 83.9 (6.5) | 84.0 (6.6) | -0.03 |
| Education (years) | <10 | 777 (10.3%) | 104 (9.6%) | 881 (10.2%) | -0.022 |
|  | 10~12 | 3,071 (40.6%) | 430 (39.8%) | 3,501 (40.5%) | -0.016 |
|  | >12 | 3,640 (48.1%) | 535 (49.5%) | 4,175 (48.3%) | 0.028 |
|  | Other | 72 (1.0%) | 11 (1.0%) | 83 (1.0%) | 0.007 |
| Equivalent income (million yen) |  | 2.6 (2.1) | 2.5 (1.5) | 2.6 (2.0) | -0.063 |
| Living arrangement | Living alone | 6,629 (87.7%) | 949 (87.9%) | 7,578 (87.7%) | 0.006 |
| Employment status | Unemployed | 7,113 (94.1%) | 1,028 (95.2%) | 8,141 (94.2%) | 0.049 |
| Assets (million yen) | <1 | 1,341 (17.7%) | 218 (20.2%) | 1,559 (18.0%) | 0.062 |
|  | 1~5 | 388 (5.1%) | 62 (5.7%) | 450 (5.2%) | 0.027 |
|  | 5~10 | 514 (6.8%) | 72 (6.7%) | 586 (6.8%) | -0.005 |
|  | 10~50 | 3,935 (52.1%) | 542 (50.2%) | 4,477 (51.8%) | -0.037 |
|  | >50 | 1,382 (18.3%) | 186 (17.2%) | 1,568 (18.1%) | -0.028 |
| Activities of daily living (ADL) | Not need care/assistance | 5,563 (73.6%) | 801 (74.2%) | 6,364 (73.7%) | 0.013 |
|  | Need but not have care/assistance | 972 (12.9%) | 127 (11.8%) | 1,099 (12.7%) | -0.033 |
|  | Need and have care/assistance | 1,025 (13.6%) | 152 (14.1%) | 1,177 (13.6%) | 0.015 |
| Body mass index (BMI) |  | 21.5 (3.1) | 21.5 (3.0) | 21.5 (3.1) | 0.006 |
| Self-rated health | Good | 6,080 (80.4%) | 885 (81.9%) | 6,965 (80.6%) | 0.039 |
| Diseases in treatment | Have | 6,664 (88.1%) | 961 (89.0%) | 7,625 (88.3%) | 0.026 |
| Degree of need for care | Independent | 4,683 (61.9%) | 671 (62.1%) | 5,354 (62.0%) | 0.004 |
| Happiness | 0 ~ 10points | 7.7 (1.8) | 7.6 (1.6) | 7.7 (1.7) | -0.012 |
| Life satisfaction | 0 ~ 10points | 7.3 (2.0) | 7.2 (1.8) | 7.3 (2.0) | -0.037 |
| Physical health | 0 ~ 10points | 6.6 (2.0) | 6.6 (2.0) | 6.6 (2.0) | 0.028 |
| Mental health | 0 ~ 10points | 7.1 (2.1) | 7.1 (1.9) | 7.1 (2.1) | 0.009 |
| Life worthwhile | 0 ~ 10points | 6.6 (2.3) | 6.6 (2.1) | 6.6 (2.3) | 0.003 |
| Ikigai | 0 ~ 10points | 6.7 (2.5) | 6.6 (2.2) | 6.7 (2.4) | -0.012 |
| Life’s purpose | 0 ~ 10points | 6.7 (2.4) | 6.6 (2.2) | 6.7 (2.3) | -0.015 |
| Contentment with friendships and relationships | 0 ~ 10points | 6.7 (2.4) | 6.6 (2.1) | 6.7 (2.3) | -0.012 |
| ^a^ SHOP: Serviced Housing for Older People | | | | | |

| **Supplementary Table 10** Comparison of health and well-being between the SHOP^a^ group and the control group after propensity score matching | | | | | |
| --- | --- | --- | --- | --- | --- |
|  |  | Control group | SHOP group | Total | Test |
|  |  | n=7,560 (87.5%) | n=1,080 (12.5%) | n=8,640 (100.0%) |  |
| Happiness and Life Satisfaction | Happiness | 7.3 (1.8) | **7.6 (1.6)** | 7.4 (1.8) | **<0.001** |
|  | Life satisfaction | 7.2 (2.0) | 7.2 (1.8) | 7.2 (2.0) | 0.887 |
| Mental and Physical Health | Physical health | 6.4 (2.0) | 6.6 (2.0) | 6.5 (2.0) | 0.009 |
|  | Mental health | **7.3 (2.0)** | 7.1 (1.9) | 7.2 (2.0) | **0.003** |
| Meaning and Purpose | Life worthwhile | **6.7 (2.2)** | 6.6 (2.1) | 6.7 (2.2) | **0.003** |
|  | Ikigai | **6.8 (2.4)** | 6.6 (2.2) | 6.8 (2.4) | **0.001** |
|  | Life’s purpose | 6.6 (2.4) | 6.6 (2.2) | 6.6 (2.4) | 0.573 |
| Close Social Relationships | Contentment with friendships and relationships | 6.7 (2.3) | 6.6 (2.1) | 6.7 (2.3) | 0.011 |
|  | Relationship satisfaction | 7.0 (2.4) | 6.9 (2.1) | 7.0 (2.3) | 0.041 |
| ^a^ SHOP: Serviced Housing for Older People | | | | | |
